# A Complex Adaptive Systems Approach to Health Professions Education Transformation: The Case of the University of Missouri-Columbia School of Medicine and an Integrated Quality Improvement-Interprofessional Collaborative Practice Curriculum

**DOI:** 10.1177/23821205251315624

**Published:** 2025-02-24

**Authors:** Katherine Stevenson, Marcel D’Eon, Linda Headrick, Boel Andersson Gäre

**Affiliations:** 1 Jönköping Academy for Improvement of Health and Welfare, School of Health and Welfare, 4161 Jönköping University, Jönköping, Sweden; 2 12371 College of Medicine, University of Saskatchewan, Saskatoon, Saskatchewan, Canada; 3 School of Medicine, 2628 University of Missouri, Columbia, Missouri, USA

**Keywords:** complex adaptive systems, medical education, quality improvement, interprofessional collaborative practice, transformational change

## Abstract

**OBJECTIVES:**

In 2003, the University of Missouri-Columbia School of Medicine (MU SOM) initiated an integrated quality improvement-interprofessional collaborative practice (QI-ICP) curriculum as part of a larger curriculum renewal process. While exploring a different case study focused on the content of the curriculum, investigators became intrigued by MU SOM's approach to change, specifically, how complex adaptive systems (CAS) thinking may have supported sustained transformational change in curriculum across multiple health professions programs. The primary aim of this study was to elucidate the aspects of MU's experience with transformation that were grounded in CAS. A secondary aim was to explore the usefulness of a CAS-based management framework for organizational transformation using the case of curricular transformation in health professions education at MU.

**METHODS:**

Data collection involved interviews, with twelve faculty from a variety of programs, and document analysis, including previously published papers and gray literature (websites, organizational charts and planning documents, and faculty development materials).

**RESULTS:**

Using abductive analysis, we found that leadership in the health professions programs at MU, informed by earlier learning about organizational sensemaking and CAS theory, addressed all 9 of the characteristics of CAS presented in the initial framework. Additionally, systematic combining revealed the need to adjust the framework to ensure applicability to health professions education. The analysis of MU's experience also offered key insights into how that transformation happened in practice. The CAS framework adjustments make explicit the importance of common purpose and the concept of leadership as an emergent event and make it easier to apply the framework to a broader set of organizational contexts, including health professions education.

**CONCLUSION:**

The use of the adjusted framework, informed by insights from this specific case, may help health professions education programs evaluate past change efforts or plan for future change.

## Introduction

Sometimes, in the course of research, one can experience serendipity. In 2013, we turned our attention to the University of Missouri and faculty perspectives on the potential connections and distinctions among competencies related to quality improvement (QI) and interprofessional collaborative practice (ICP) in health professions education.^1^ As we developed the case focused on competency domains, we became intrigued about how, in fewer than 10 years, the faculty had managed to initiate and sustain an integrated QI-ICP curriculum across multiple health professions programs. In our experience as faculty in 3 different institutions, change within multiple curricula in the complex environment of health professions education can seem impossible. Further, while analyzing the original data set that included faculty perspectives on how to integrate QI and ICP curricula, KS noted threads of complex adaptive systems (CAS) thinking. As part of an interactive research process, these threads were confirmed by a participant to be deliberate. While not intentionally employing a fulsome framework, formal educational leaders were learning about the concept of organizational sensemaking in the context of CAS^2^ and trying to apply it to the change they were seeking. Thomas suggests that “questioning and surprise, intelligent noticing and serendipity”^3^ (p. 579) are key to phronesis-based case study research. Phronesis is defined as “practical wisdom, or knowledge of the proper ends of life, distinguished by Aristotle from theoretical knowledge.”^4^ We took this opportunity to explore the practical wisdom of the faculty involved in curriculum change at the University of Missouri, curious if we might be able to propose a provisional CAS-based framework to support transformational change in health professions education.

## Background

Curriculum change has been likened to “moving a graveyard,” and large-scale curricular change might be described as transformational. While some argue that “transformational” is overused or not well defined, Chapman offers the perspective that transformational change can be defined by the type of change sought.^5,6^ Drawing on the concepts of first- and second-order change, she argues that attempting to completely redesign a system requires work on changing values, beliefs, and attitudes as a precursor to change of systems and processes and, therefore, can be described as transformational.^6^ The challenge of integrating QI and ICP content into health professions education can seem on the surface to be about process, systems, and structure, but educators who have tried to integrate one or both will recognize that it is largely about shifting the attitudes, beliefs, and values of people.^7–9^ Additionally, the context where the change is taking place can increase the complexity of the change. Higher education represents an organization of organizations, so to speak, with academic fields and expertise intersecting, that is, colleges, schools, or departments in the context of a specific academic institution. Higher education focused on the development of health professionals is further complicated by the number and variety of professional groups, the power dynamics among them, and the intersection of the academic setting with clinical healthcare delivery institutions.

Setting aside the transformational debate, change has often been conceptualized and approached as either planned or emergent. Planned change assumes linearity, and many frameworks for planned change, including Kotter's 8 steps and the ADKAR model, can be situated in Lewin's classic model of Unfreeze-Move-Refreeze.^10–13^ In contrast, an emergent approach emphasizes that “change should not be perceived as a series of linear events within a given period of time, but rather as a continuous, open-ended process of adaptation to changing circumstances and conditions”^14^ (p. 497). Kezar argues beyond the either-or of planned and emergent and suggests an analysis of higher education change initiatives should use a multifaceted framework, including evolutionary, social cognition, cultural, political, management science, and institutional lenses.^15^ The need for a multifaceted framework reinforces the complexity inherent in institutions of higher education and in the people who must enact the changes. With complexity in focus, CAS theory may provide us with an approach to better understand and manage change.

CAS has been recognized as potentially comprehensive and integrative enough to study educational change in higher education,^16,17^ albeit most of the application to date has been in primary and secondary education.^18–21^ Given that academic institutions and systems have been characterized as being comprised of “multiple layers of both hierarchical and collegial networks that seem at times to stubbornly resist transformative change, while simultaneously adapting and evolving”^22^ (p. 121), we argue that health professions education is also a CAS. Using a CAS lens to study the example of a system that has quickly and successfully integrated a longitudinal quality improvement and interprofessional collaboration curriculum can help us better understand how to effectively lead whole system curricular change in health science programs, no matter the content.^23–26^ While there are a handful of examples of CAS frameworks used in organizational change in the context of health services^27–29^ and higher education,^22^ to the best of our knowledge, there is no CAS-based framework specific to change in health professions education. The primary aim of this study was to elucidate the aspects of MU's experience with transformation from 2003 to 2013 that are grounded in CAS. A secondary aim was to explore the usefulness of a CAS-based management framework for organizational transformation using the case of curricular transformation in health professions education.

## Methods

This research study formed part of the first author's doctoral dissertation. Due to life circumstances impacting KS, including sequential and multiple parental and bereavement leaves, there was a 10-year delay between data collection and final analysis and reporting. It is important to note that literature reviews were completed at intervals during the delay, and these reviews affirmed the continued relevance of the study. Ethical approval was maintained throughout this entire period.

### Context

In 2003, the MU SOM embarked on a renewal process called the “MU 2020 Initiative” whereby they clarified MU's medical education mission to educate physicians, “to provide effective patient-centered care for the people of Missouri and beyond” and established 8 “key characteristics”^23^ (ps. 310) that would describe their medical school graduates (Table 1). Early in the MU 2020 Initiative journey, faculty members initiated a combined QI-ICP education program. Over the decade in focus, they partnered with faculty from other University of Missouri health professional education programs and progressively developed curricular improvements to teach health professions learners both how to work together in interprofessional teams and how to continuously improve their work through the application of quality improvement methods and tools. A key component of their journey was participation in a faculty development program for interprofessional team-based care offered by the Macy and Hearst Foundations,^30^ which they often referred to as the “Macy Grant.” As one of 8 participating institutions, they had the opportunity to learn together in a small interprofessional faculty team and were also invited to deliver teaching on topics, specifically QI as a vector for interprofessional education and change management. Not only did they develop and evaluate their innovations in teaching, but they also published extensively.^31–35^ MU SOM has been recognized for its efforts with an Association of American Medical Colleges Learning Health System Challenge Award.^26^

**Table 1.** MU 2020 initiative. Eight key characteristics of graduates from MU SOM.^23^

Able to deliver effective patient-centered care
Honest with high ethical standards
Knowledgeable in biomedical sciences, evidence-based practice, and societal and cultural issues
Critical thinkers and problem-solvers
Able to communicate with patients and others
Able to collaborate with patients and other members of healthcare team
Committed to improving quality and safety
Committed to lifelong learning and professional formation

### Contextual framework

As described in the background, the application of CAS to organizational change has been limited in higher education. Additionally, previous literature has not, to our knowledge, offered specific management practices to support leaders to engage in change from a CAS perspective. One advantage of the delay between data collection and the final analysis of this study was the publication, in 2023, of a practice-focused CAS-based framework. Discovered in the course of updating the literature review, the proposed framework from Riaz, Morgan, and Kimberley is based on 9 characteristics of CAS (Table 2) and integrates theory related to both organizational transformation and CAS, offering not only a reflection on theory but also concrete management practices.^14^ The framework from Riaz et al. is potentially useful for both retrospectively understanding transformative change and prospectively leading it; we have therefore chosen it to be in focus throughout our analysis.

**Table 2.**
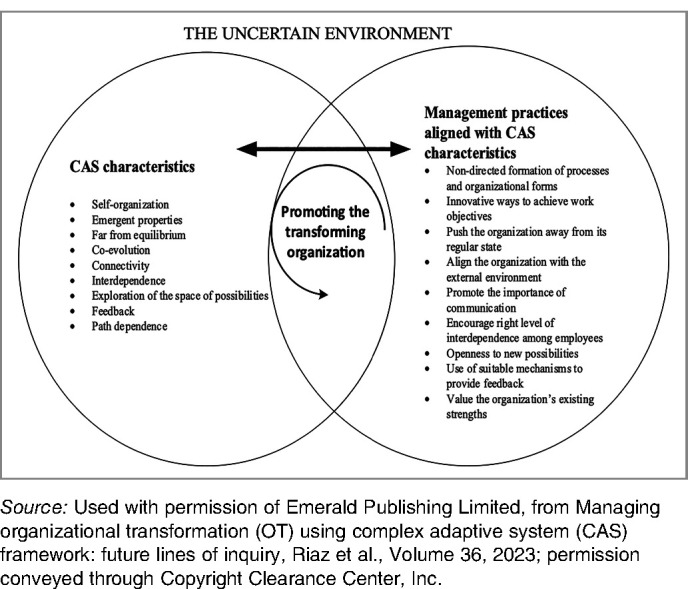
Initial framework by Riaz et al. Management practices based on CAS theory to support transformational change.^14^

### Study design

We used a case study approach underpinned by an interactive research model, with the case being efforts to implement an integrated QI-ICP curriculum at the University of Missouri-Columbia School of Medicine (MU SOM) between 2003 and 2013. In the tradition of action research, interactive research is “characterised by a continuous joint learning process between the researcher and the participants”^36^ (p. 233) where the distance between the researched and the researchers is often diminished. LH, a faculty member at MU, did not participate in data collection or analysis but contributed to the interpretation and writing process. The reporting of this study conforms to the SRQR guidelines (see Appendix A).^37^

### Participants

With the goal of gaining insight into the case^38^ and in alignment with qualitative case study methodology, we used purposive sampling.^39^ With the guidance of MU academic program leaders, we selected and invited 19 academic and clinical faculty from diverse backgrounds who had experience in leading and teaching within the interprofessional quality improvement education program from 2003 to 2013.^40^ In total, 12 faculty agreed to participate in data collection, with representation from medicine (7), nursing (3), pharmacy (1), and health care administration (1). Of the 12, 5 participants had roles primarily within an academic unit (University of Missouri), and 7 had roles that were predominately in clinical education (University of Missouri Health Care). In addition, 7 had roles connected to an academic-clinical bridging organization, the Center for Health Quality at the University of Missouri.

### Procedure and data collection

Qualitative data in this and our former study^1^ included faculty interviews. This case also incorporated qualitative data extracted from previously published papers and gray literature (websites, organizational charts and planning documents, and faculty development materials).

Data were collected during a 1-week period in January 2013. In alignment with the case study methodology,^41^ KS conducted initial one-on-one interviews with 11 of the 12 participants using a semistructured guide, field-tested elsewhere (not published). Interviews focused on faculty perspectives regarding QI and ICP content, delivery methods, and rationale of teaching choices. KS conducted an observation of teaching practice and then a second interview (*n* = 6) within 7 days of the initial interview using a modified stimulated recall technique. By anchoring an interview in an observation of practice, stimulated recall is designed to uncover thought processes and beliefs.^42^ In this study, stimulated recall supported reflective practice through reflection on action, prompting respondents to “make explicit and articulate the thinking, knowledge, theories, and beliefs that guided their teaching practice”^43^ (p. 290). Due to concerns that recording teaching might interfere with student learning and/or not be feasible due to teaching modes (eg, simulation), we modified the typical approach to stimulated recall of using video recordings and instead anchored the interview in notes from observations of teaching sessions and teaching materials. We identified an additional participant in the course of observations, and so one participant was involved in only a stimulated recall interview.

Documents that were included in the inductive analysis were collected through the course of interviews, a search of peer-reviewed and gray literature that included descriptions of strategic and curricular interventions, and the MU website. All interview data, including stimulated recall, were collected in January 2013. Data from both the initial and stimulated recall interviews were transcribed and analyzed with the assistance of QSR NVivo 10 software. Document-based materials were analyzed and grouped into themes manually.

### Analytic approach

In alignment with an interactive single case study, we used an abductive approach to data analysis. Rather than a linear deductive or inductive approach, abductive analysis is iterative, using “a back-and-forth process between the research evidence and considerations of theory”^44^(p. 305). Abduction is particularly appropriate to interactive research in that it doesn’t need to manage positionality but rather sees strength in the researcher entering the field with as much prior knowledge as possible.^45^ All 4 investigators had practice experience in health professions education. Two as physicians (LH and BAG) with leadership experience in QI and ICP education, one with a doctorate in education (MD) with a focus on ICP education and one (KS) as a doctoral student with a clinical background in physical therapy and over a decade of experience teaching QI and ICP.

### Analysis

We used systematic combining, a specific approach to abductive analysis that “extends the interplay among research activities to include the role of theory and framework”^46^ (p. 1279). Systematic combining includes matching and redirection, which are 2 related, iterative, and non-linear steps. Matching is about “going back and forth between framework, data sources, and analysis”^45^ (p. 556). Redirection supports matching and involves (1) the use of multiple sources of data and (2) a “tight and evolving framework”^45^ (p. 558) and theory to stimulate further curiosity and discovery.

**Figure 1.**
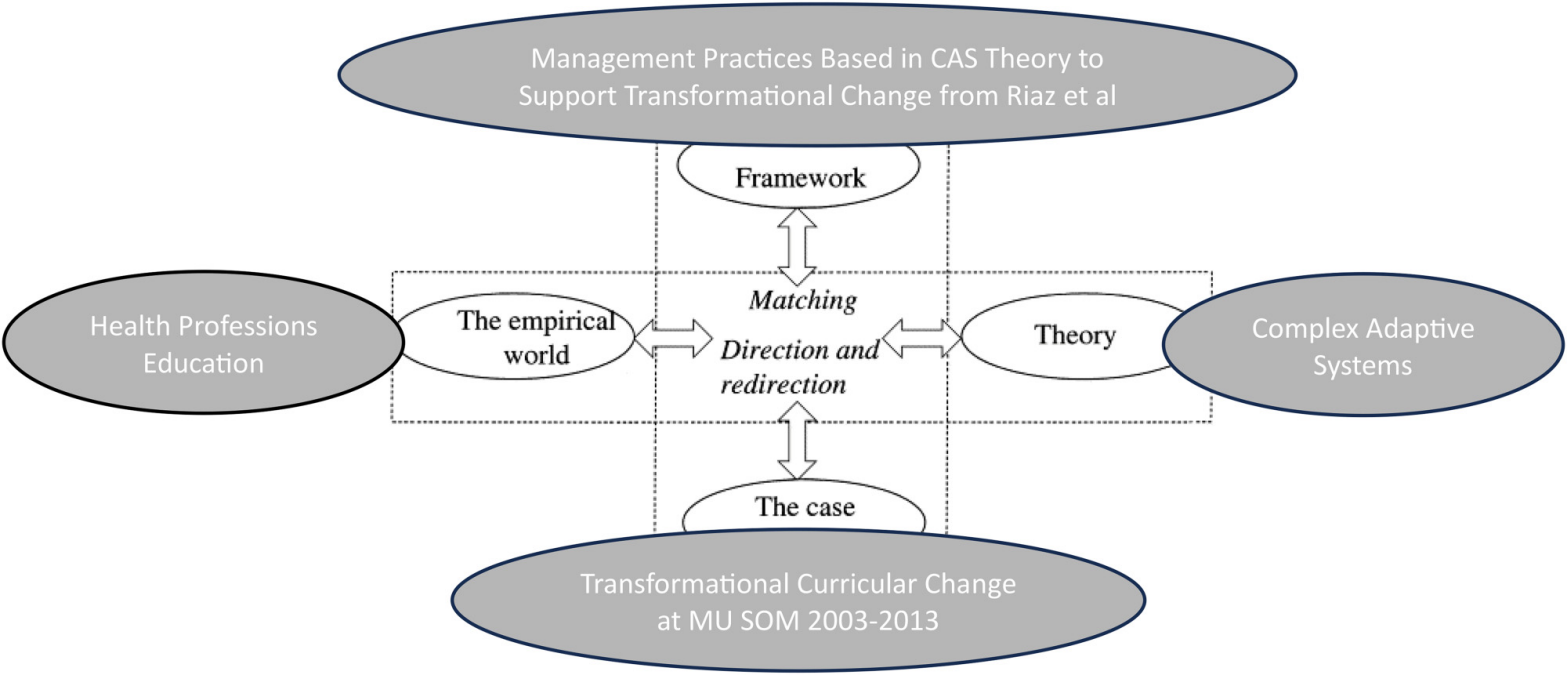
Visual description of our approach to systematic combining adapted from Dubois and Gadde.^45^

*Source:* Used with permission of Elsevier Science & Technology Journals, from Systematic combining: an abductive approach to case research, Dubois, Anna; Gadde, Lars-Erik, Volume 55, 2002; permission conveyed through Copyright Clearance Center, Inc.

Our analysis involved familiar qualitative processes like coding and theming, but the data were returned too frequently, with both the theory of CAS and the Riaz framework^14^ in mind. The framework adjustments evolved as themes emerged from the data and by considering the whole of the case and CAS theory. Figure 1 depicts our approach to systematic combining.

## Results

Related to the primary aim, we found that leadership in the health professions programs at the University of Missouri, informed by earlier learning about organizational sensemaking and CAS theory, addressed all 9 of the characteristics of CAS presented in the initial framework suggested by Riaz et al. (Table 2).^14^ Additionally, related to the secondary aim of exploring the usefulness of the Riaz framework, through the use of systematic combining, we identified the need to adjust the framework by (1) refining 4 of the 9 existing management practice descriptors and (2) adding one new CAS characteristic and 2 related management practice descriptors so that our adjusted framework has 9 CAS characteristics and 11 management practices. We will start by describing the adjusted framework itself, including reference to theoretical grounding for the additional characteristics and management practices. Then, we will report our complete results in the context of the adjusted framework.

### The adjusted framework

While the management practices described by Riaz et al^14^ speak to the role of formal leaders, our familiarity with CAS theory and emerging themes from the empirical data provoked us to challenge the absence of leadership as a process within the framework. Going back into the literature specific to complexity leadership to inform our analysis,^47,48^ we added leadership as an emergent event as a feature of a CAS.

Adjustments to existing management practice descriptors were informed by CAS theory, data from the case, and considerations of applicability to the empirical world of health professions education. First, the management practice related to the non-directed formation of processes, and organizational forms was clarified by adding reference to a common purpose. Second, the management practice related to the achievement of objectives was expanded by removing the specificity of “work” to more broadly encompass learning. Third, given the structural complexity of the health professions learning context, where multiple professional programs interact with each other and the clinical delivery environment, the practice related to alignment of the organization was enlarged to include alignment of both the internal and external environments. Fourth, to be more inclusive regarding who might be engaged in interdependent relationships, we substituted “agents” for “employees.” Finally, related to complexity leadership theory and themes in the data, we added 2 new management practices: supporting a collective identity and nurturing productive tension. We also edited the grammar to ensure that all practices were framed as actions.

### Results in the context of the adjusted framework

Results from the case (including representative quotes, in italics) are now presented using the adjusted framework described in Table 3, focusing specifically on the management practices. Illustrative quotations derived from participant interviews are anonymized by assignment of a letter from A to L. Document-based data, whether published or not, are referenced inline.

**Table 3.** Adjusted framework informed by systematic combining (*additions* and deletions).

CAS CHARACTERISTICS	ADJUSTED MANAGEMENT PRACTICES
Self-organization Emergent properties Far from equilibrium Co-evolution Connectivity Interdependence Exploration of the space of possibilities Feedback Path dependence *Leadership as an emergent event*	*Use* non-directed formation of processes and organizational forms *toward a common purpose* *Support* innovative ways to achieve work objectives Push the organization away from its regular state Align the organization *internally and* with the external environment Promote the importance of communication Encourage the right level of interdependence among *agents employees* *Stay* openness to new possibilities Use of suitable mechanisms to provide feedback Value the organization's existing strengths *Support collective identity* *Nurture tension to create adaptive change*

#### Use non-directed formation of processes and organizational forms toward a common purpose

Senior leadership at MU SOM created an environment supportive of self-organization, specifically by ensuring clarity of common purpose. The common purpose is embodied in the 8 “key characteristics” defined at the outset of the MU 2020 Initiative (Table 1).^23^ The key characteristics remind everyone of the end goal without being directive about *how* to get there. Instead of overspecification of teaching processes, faculty were supported with “simple rules” or minimum specifications that provided some boundaries but also a great deal of freedom.

*… teaching quality and safety skills [and] interprofessional teamwork, or preparing faculty to do that, …, is complex activity. It's not complicated; certainly not simple. And that means you need simple rules to govern it. [For example], learning has to be experiential, and it has to be facilitated by somebody who knows what they’re doing. (E*)

Additionally, while there were many formal opportunities and structures for the learning and development of faculty, there was also a culture encouraging everyone to learn skills in QI and ICP on the job with supportive mentorship.

*it's been…experiential learning for me, … so not something I had formal training or background in, but just the good fortune of [experiencing] roles that you have no formal education or background in because you are interested and there are those around who can mentor you along your way. (J)*

#### Support innovative ways to achieve objectives

MU SOM senior leaders nurtured grassroots and mid-level leadership to engage in curricular innovation, being attentive to balancing the academic and care delivery context and so including those who had a foot in both worlds. Through innovation, faculty were able to achieve learning and work-related health system objectives simultaneously. For example, they “successfully involved a large number of medical and nursing students by identifying a focus – the prevention of falls – that contributed to the health system's clinical quality improvement and patient safety efforts”^24^ (p. 2675).

MU SOM sought voluntary engagement of representatives from multiple healthcare professions who could act as effective role models for the interprofessional and quality improvement mindsets that they were seeking to develop in learners.

*I think a key … was getting some original leaders [voluntarily] involved that had a role, both in the care delivery system and at the school of medicine and were able to kind of bubble that up from the grassroots or from the middle leadership level. That wasn’t a top-down decision. (E)*

Individual faculty members also collaborated within the institution to innovate on teaching methods and approaches. An example was engaging health system interpreter service staff as standardized patients in case-based learning about cultural humility.

*The [case] that we did today is …[about] culture humility…the twist to this one is we use our interpreter service (in the role of standardized patients), and they are very happy to educate a whole group of healthcare providers in a very unique way. (F)*

Innovation also included attempts to integrate competencies in initiatives like the “Legacy Teachers” program, which continues to this day.^49^ In this program, MU SOM flips the classroom in a patient- and family-centered way by having third-year medical students submit an essay, poem, or artwork honoring a legacy teacher, that is, a patient that they have cared for and who has “had a lasting impact on the students’ ability to provide patient-centered care”^25^ (p. 167). Objectives for the program address competencies related to patient-centered care, communication, lifelong learning, and professional formation.^23^

#### Push the organization away from its regular state

As a team, the Missouri faculty and leadership enrolled regularly in external learning opportunities. These shared experiences allowed them to see new possibilities and kept the system on the edge of change. For example, the Macy Grant Interprofessional Training Week in 2012 allowed for learning as a faculty team. This opportunity aligned with the organizational goal of integrating QI and ICP education, including a full workshop session on “QI as a vector for IPE” which incorporated improvement knowledge content in sessions on change management.^50^

#### Align the organization internally and with the external environment

There is an aspect of Missouri's work that involves alignment with the external environment of quality and safety science. For example, Missouri initially had a very strong focus on safety, clearly influenced by initiatives like the Institute for Healthcare Improvement's 100 000 Lives campaign and landmark reports like the Institute of Medicine's Crossing the Quality Chasm and To Err is Human.

*It started about ten years ago, focused very heavily initially on error disclosure but …maybe one year into it [we added] some principles of systems-based care and providing students with fundamental tools of quality improvement after they were sort of introduced to … the “quality chasm”…. (H)*

Over time, as safety science progressed, concepts like the “second victim” were included.

*And so, he knew the term second victim and he told me we needed to figure it out… (I)*

Similarly, topics like health literacy and cultural humility were added to the understanding of what it means to deliver high-quality patient-centered care.

Given the propensity for a lag between best-known practice and actual practice, educators had the additional challenge of aligning to the clinical setting, staying alert to the potential conflicts between what was being taught and what was being modeled. For example, while supporting learning about systems thinking and safety, they had to prepare learners for a context where sometimes there was still a focus on individual blame.

*We’re in such a hybrid between the new world of blame-free, focusing on systems … versus the old model … I can go to units right now that are real name, blame, shame, … and I could go to other higher performing units where … people are working with each other, interacting with our managers and talking and as a team. (I)*

In terms of aligning faculty and learner contexts, the leadership also created embedded internal opportunities. Specifically, in one part of the curriculum where interprofessional student teams tackled an actual improvement project, they paired clinical faculty and staff who had limited experiences with QI with senior learners.^32^

*… the staff are taught by example of participating in quality improvement…[we] pick some faculty to participate… who haven’t done a lot of QI, …, and that could use either a refresher course or they just need some basics. (B)*

This approach allowed for simultaneous development of learners and novice staff and faculty (from a QI perspective) in interprofessional quality improvement, while also supporting the more expert faculty to deepen their skills in mentorship. As a result of their participation, many of the faculty stayed involved, teaching and focusing on the quality of their work.

*Most of them are still involved in quality improvement. Many of them are helping to teach this stuff. Now many of them have gone on to build quality into their career (E)*

#### Promote the importance of communication

Missouri blended formal and informal structures to support connectivity among faculty and staff. The Center for Health Care Quality (CHCQ) acted as a bridging organization not only between academic and clinical environments but also between departments within each of these environments.

*Typically, if you’re an academic and have a center, … your goal is to create as big an island with as steep cliffs, as many barriers to intrusion see you have as much autonomy to do what you want to do … my vision is you need something that's going to be a causeway that connects the islands. (C)*

In guiding documents for the CHCQ, these relationships were made visible through an organizational chart and project management documentation that made clear the contribution of the teaching, research, administrative, and consultative arms to the strategic goals of both the School of Medicine and University of Missouri Health Care.^51^

MU leveraged informal structures as well. Key organizational members pulled various partners into discussion, including leaders of academic curriculum blocks and clinical experiences, and created cross-functional spaces to work through ideas and test them.

*There isn’t really any formal structure holding the whole group together…[but] they have legitimacy. And they have that because of their content expertise. And they also have positional expertise in that I lend my support and [other leaders] lend their support. (D)*

Connectivity as seen through an emphasis on the importance of communication was explicitly promoted among learners. Three of the 8 key characteristics are tied to communication skills, that is, first to “able to communicate with patients and others,” to “effectively collaborate with patients and other members of health care team” and so “deliver effective patient-centered care.” Additionally, in 2010, MU SOM added an evaluative component whereby medical students must pass a Patient-Centered Care Objective Structured Clinical Examination in order to graduate.^23^

#### Encourage the right level of interdependence among agents

MU SOM education leadership set up faculty and learners in interprofessional and cross-functional teams when engaged in teaching and in doing improvement work, including formal partnerships with the MU School of Health Professions, the MU Sinclair School of Nursing, and the University of Missouri-Kansas City School of Pharmacy. The SOM approach to teaming was instrumental, ensuring that the “themes of patient safety, quality improvement and team-based care delivery [were] interwoven throughout the 4-year curriculum through a variety of interprofessional learning opportunities ranging from error disclosure training to training in interprofessional communication”^25^ (p. 166). The work culture further inspired mid- and lower-level leaders to role model interdependence where clinically trained faculty and staff demonstrated explicit appreciation for administrative and support staff roles in improvement and collaboration.

*…when you go out into the world of work and in the teams … those teams are not all clinical. And the clinicians and the administrative types, and the support system …cross and need to talk. (C)*

*…I realized that the front line really understands how to get the work done and they haven’t figured out how to speak up. And so…I’m going to gather people by the old oak tree. I’m going to make friends of all the … workers, …so when I blow a whistle some day when I really need them, they’re all going to gather and we’re going to change the world basically. (A)*

Peer teaching was emphasized in many of the programs, internally and externally, among both faculty and learners.

*This is about peer teaching. So, if you know more, you [teach] it to your peers… [and] you bring an entire interprofessional group's knowledge of safety to a much higher level. (F)*

In the Macy Grant Interprofessional Training Week, an MU faculty member led sessions for faculty participants specific to managing change using QI as a frame.^50^ For pre-licensure students, peer feedback was encouraged to build a sense of dependence on each other as learners rather than relying solely on the educator.

*Because they have to respect and trust each other. And if I (as an educator) correct them, then I become the source of information. If [learners] correct each other, they’re responsible for their own learning and the boundaries of that learning. (D)*

In project-based courses, where students worked on real problems, they presented their results to leaders in the system with an opportunity to sustain their work beyond the course.

*…the leaders pick the projects and then they’re in the audience when the solutions are delivered. And the people work really hard because they know who the audience is at the end too. (K)*

#### Stay open to new possibilities

A group of faculty and health system leaders who were very interested and enthusiastic about improvement and interprofessional collaboration were placed in key positions in the early 2000s. This enthusiasm led to a strategy to develop QI and ICP capacity. Over the course of about 6 years, they went from a small number of enthusiasts to almost 60 trained medical faculty.

*… I could have counted on one hand probably the clinical leaders in 2002 that knew how to really use quality improvement tools to do a project and to use them appropriately…compare that to where we’ve got four or five dozen docs walking around. (E)*

This focus on development stimulated possibility and exploration in an ever-widening group of interested faculty and staff. There was also a general attitude of being open to evolving the curricula as emerging opportunities presented themselves; learning experiences in other programs through initiatives like the Macy Grant provoked continued development.

*…we adapted something that the University of Washington was doing in Seattle and they used faculty and we introduced standardized patients… and it was a gap that was maybe not covered as fully as we could. And so, I think that's why the curriculum has seen some evolution over time. (H)*

Importantly, the mission and key characteristics of the School of Medicine created a strong sense of purpose that guided and challenged faculty at all levels. Using formal and informal feedback to make visible whether educational processes were having the intended effect, especially as it related to QI and ICP, kept the system open to improvement and encouraged modeling of “commitment to improving quality and safety”^23^ (ps. 315).

*…if we’re seeing a behaviour in our students that is unanticipated, then part of what I like to do is step back and say what are we doing to drive that behaviour. What messages are we sending? So how does [interprofessionality], how does quality and safety become a part of what the students routinely expect to do, are routinely measured against, and that we send a very clear message that that is an expectation beginning on day one. (D)*

#### Use suitable mechanisms to provide feedback

As a higher education system, the faculty at Missouri relied heavily on feedback from learners to guide changes to the curriculum. Feedback came not only in the form of ubiquitous course evaluations but also through assessment of learner performance such as applying tools like the Objective Structured Clinical Examination (OSCE) to patient-centered care as a key dimension of quality,

*It's about what they can actually deliver… It's about what they can do in the room with the patient when they’re under stress. And that's what the [patient-centered care focused] OSCE evaluates. And I think that's where this field needs to go. (D)*

Evaluation data were also collected through focus groups,

*The next session … is a little bit difficult for the students to understand because it's about quality improvement…we changed it three times now and we’re going to change it one more time because…I’ve had a focus group with a couple of the students. (F)*

At every stage of learning, reflective practice was used as a source of feedback to support personal improvement and development for both learners and faculty.^23^

Program leadership also paid attention to evaluation results over time and what they meant for the program, not just for individual courses. Longitudinal data led to a “corporate strategy” that enabled informal and formal leaders to teach and role model both interprofessional and quality improvement skills and values in clinical and academic settings.

*What we hadn’t anticipated though is that when [we] remeasured those a year later, that some of the [ICP] attitudes that had improved were right back where they started…and so in 2004 is really where we started saying okay what is our corporate strategy for developing capacity. (E)*

This meant applying an improvement approach to the curriculum itself. For example, while participating in the Retooling for Quality and Safety program in 2009-10, faculty ran 6 improvement cycles of an interprofessional learning activity, using “faculty and student feedback to evaluate the experience and revise it for the next group of students”^24^ (p. 2673). In this same initiative, feedback gleaned from students who were in the midst of taking the course led to improvements in the standardized form used to support an interprofessional assessment of risk of falls in patients.^24^

#### Value the organization's existing strengths

In 1993, 10 years prior to the launch of the MU 2020 Initiative, MU School of Medicine introduced a significant change by moving to a problem-based learning (PBL) curriculum.

*It was amazingly challenging (transitioning to a PBL curriculum). I mean I had people in my office all the time. What if [a student] gives misinformation? I said they’ll self-correct. We’ve taught them how to do that. (D)*

Shifting to PBL was determined to be successful with medical students scoring “far above their peers in medical licensing exams and in subsequent residency program director reviews”^25^ (p. 166). One participant also described how students who learn in the PBL curriculum are more prepared for the QI-ICP curriculum.

*I would say that medical students that are part of a PBL curriculum … would be more open to interprofessional collaboration or learning… certainly the same openness to learning from others and to opinions other than your own and to being challenged or being… able to accept both praise and … questions or even potential challenges… (H)*

The use of PBL in the medical curriculum was leveraged as an opportunity to teach quality and safety concepts alongside basic science within the PBL curriculum framework; although, at the time generally in medical education, interprofessional learning of basic science concepts was absent.

#### Support collective identity

At MU SOM, formal leaders recognized the distinction between leaders and leadership and how leadership emerges in teams. When asked to describe their approach to leadership development, one participant made the distinction that they support an “institutional leadership development program, not an individual leadership development program” (C) and consider the development of networks and team connections in this context.

*If you think about just learning to work in a team, and the challenge of a team, the logistics, and the communications –… how would you get a group headed in the same direction? And particularly when there's not a designated leader. (C)*

Through the shared experience of learning together and developing and testing changes to curriculum together, faculty at MU, no matter their department, spoke as a collective “we.” The formation of this collective identity was most visible in the results from faculty who spanned clinical and academic contexts, that is, contributed to health professions education from their clinical positions and those working primarily at a satellite campus with a foot in essentially 2 health professions education programs. For clinically based educators, the “we” was a collaborative team that was inclusive of academic program leaders to whom they would provide real-life QI projects and from whom they would request certain content be included in the curriculum.

*So there's [a] need [in the clinical context of medical training] and …we …contact the [academic] program directors and say these are things we would like to be in that curriculum and as part of that we have this wealth of expertise in our med-neuro ICU critical care nurses… we look for way to get at the didactic needs, but also to frame that within collaboration. (J)*

For the faculty members who spanned sites, there was the “we” connected to MU SOM where the focus was on the development of the QI-ICP curriculum and the “we” of the satellite campus where learning was uni-professional for the most part. This participant spoke at length about trying to connect the 2 contexts, exposing colleagues to what was possible in the environment created by MU. The following fully de-identified quotation illustrates this fluid sense of identity as the faculty member, who is based in Kansas City, switches from we to they:

*And so when **we** (referring to themselves as part the Kansas City faculty) started getting invited to do things here (Columbia) … now **we** (referring to the Columbia faculty) are telling Kansas City, “Hey, we’re doing this, and we started something new in medical error disclosure and things of that nature”…And so interestingly enough in Kansas City, there's all the same disciplines…and **they** (referring to Kansas City) actually don’t come to the table together very often. It's a different culture than it is here (Columbia).*

Due to the limited number of faculty who were based in Kansas City and the risk of being able to identify which other data were theirs, we’ve chosen to not indicate the specific participant responsible for this quotation.

#### Nurture tension to create adaptive change

Tension in service of adaptive change was somewhat naturally supported in that a curriculum transformation focused on QI and ICP required the bringing together of a diverse group of faculty. The CHCQ was a formal structure that pulled together the diverse goals of improvement, education, and research and included representation from academic and clinical contexts and multiple professional perspectives. Formal leaders also ensured that faculty learning around ICP and QI happened in interprofessional groups. Tension was generated in 2 fundamental ways. First, constraints regarding resourcing and time protection were clear. Much of the curricular innovation required clinicians willing to volunteer their time and faculty willing to coordinate logistics and work in the absence of dedicated administrative support or protected time,

*When I have leaders, particularly physician leaders on improvement teams, they are donating their time. They don’t get paid. (A)*

Second, application of an improvement mindset to the curricular innovations themselves drove some level of uncertainty through a habit of trying new approaches to teaching and learning,

*we’ve done some… experimenting with it too, and if the students are completely unfamiliar with the subject matter … then that can be good because it helps with the brainstorming,[but] if they have had some background, it helps them to be a little more specific about what they are talking about. (L)*

Being open to experimenting was also risky and made challenges visible in terms of less-than-optimal student experience or learning, with faculty sometimes encountering difficult lessons along the way,

*I went through a fair amount of emotional rollercoaster with that course during the three years …I thought I was pretty smart. I made some changes and some of which did not work very well, and it was because we engaged everybody except the medical students. (E)*

## Discussion

Examining the journey of MU's School of Medicine using the Riaz framework elucidates the CAS aspects of MU's experience, making those lessons accessible to others. At the same time, the MU experience provides evidence of the usefulness of the framework and identifies adjustments that broaden the framework's applicability. As one might expect, with CAS in mind, the change approach was both planned and emergent. One can identify tactics used by MU SOM across the continuum of innovation proposed by Greenhalgh et al from “let it happen” (emergent) to “help it happen” (social) to “make it happen” (managerial), with the bulk of their work leaning toward sensemaking and emergence.^52^ The MU 2020 Initiative, led by faculty who had CAS in mind, created a clear and shared common purpose that ensured focus for the changes while “the simple rules approach” allowed for creativity and flexibility. While the outcomes of the MU 2020 Initiative in terms of mission and the 8 characteristics were established in 2003, they have stood the test of time and remain unchanged to this day, confirmed at a recent review in 2021.^53^

We can’t overstate the importance of leadership at MU, especially when conceptualized not as a role but with a view to complexity, and so added both a characteristic and management practices related to leadership to the framework. In CAS, leadership is “seen not only as a position and authority but also as an emergent, interactive dynamic”^48^ (p. 299). It should be “understood as the capacity to influence others…[and] can be enacted within every interaction between members”^54^ (p. 618). The implication is not that formal leaders don’t matter (MU had strong and visionary leaders), but that everyone in the system has the capacity to enact leadership in the service of change. MU successfully supported a collective identity, that is, a sense of “we”, despite different departments or even sites. This identity strengthens over time as agents work toward a common product or purpose.^47^

Productive tension in the context of emergent leadership occurs when the interactions between agents spark exchanges that lead to adaptive change.^47^ As opposed to adaptive change, which is more technical or skill-based, “adaptive change is systemic, … requiring a fundamental reappraisal of beliefs and values in an organisation or a change of organisational culture”^55^ (p. 360). Formal leaders can foster productive tension by leveraging diversity as a design principle and inject tension by issuing a challenge or constraint to a team.^48^ The concept of adaptive change with its emphasis on re-examination of values and beliefs resonates strongly with the description by Batalden and Foster of the next phase of improvement work, Quality 3.0.^56^ The focus of their work is on the transformation of the relationship between the delivery system and the patient, to where “both ‘relationship’ and ‘activity’ are fundamental [and]… they are connected by knowledge, skill, habit, shared power and a willingness to be vulnerable on the parts of both parties”^56^ (pii12). We would argue that this evolution could also apply to team dynamics in the context of clinical ICP or educational ICP in service of adaptive change.

MU SOM's history of curricular transformation around PBL was key in supporting success with this more extensive change work. Experience with such a significant and successful change in teaching approach likely contributed to faculty confidence with ongoing change and improvement. The histories of the organization and the people with past change “are very important, … because development options can be preferred (and possibly locked-in or out) influencing option choices available for future actions”^57^ (p. 914). While we focused on only one key past change with PBL, it has been found that the “perceived quality of past change experience might be more impactful than the number of previous changes”^58^ (p. 1191). In MU SOM's case, key leaders involved with the PBL change were also involved in the work to integrate QI and ICP education. The continuity of leadership in change may support what Thurlow and Mills argue as being key to developing “narratives of legitimacy,”^59^ (p. 249) when key figures can tell the story of the history and experience of change and therefore “contribute the shared meaning of change within the organization”^59^ (p. 247). Honoring the organizational history with change connects well with the concept of “organizational change capability” or OCC from the management literature. A model still in development, proponents of OCC argue that it is through attempting change and learning from both successes and challenges that organizations can develop “capabilities to initiate and implement change”^60^ (p. 779). The key here is the word “learning.” OCC researchers have found that organizations that “promote an environment conducive to developing knowledge and skills are more likely to have a greater capability to change due to the organizational plasticity acquired by learning mechanisms”^58^ (p. 1192). As demonstrated in our study, faculty deliberately built in feedback loops that supported learning as they implemented changes.

While not explicit in the framework offered by Riaz et al.,^14^ the concept of self-similarity is present in this case as a through line. Self-similarity has been identified as a key feature of a CAS and means that at various levels of granularity, structures, and processes replicate.^61^ In nature, you can see this exhibited structurally through fractals, for example, the shape of ferns, snowflakes, or tree branches.^62^ In social science applications of CAS theory, self-similarity has been linked to organizational identity, which in turn is linked to purpose and heavily influenced by path dependence.^61^ In the case of MU SOM, we can see self-similarity as “fractals of learning” and development. The curricular transformation toward an integrated QI-ICP curriculum was itself a large-scale QI-ICP project, pulling in principles of QI in the iterations and evaluation of new curriculum and ICP in how faculty worked together and across silos. Such an emergent approach stands in stark contrast to traditionally linear approaches to large-scale change implied in many change and implementation models. Interestingly, while Kotter's original 8-step model is still used in healthcare and health professions education, Kotter himself has recognized the limitations of linear, implementation-focused approaches in his development of accelerators and his latest work on change that focuses on uncertainty.^63–67^ The MU SOM approach to change, based on an understanding of CAS, allowed them to embody QI and ICP from the macrosystem perspective while aiming to integrate QI and ICP into health professions learning in the meso- and microsystem.

## Methodological considerations and future research

The use of an interactive case study and an abductive analytic approach provided a foundation for exploring the experience of MU SOM from 2003 to 2013 in the context of CAS theory and a related management framework. While there were 10 years between data collection and analysis, the delay serendipitously and fortuitously allowed for the use of a more recently published practice-focused framework in the analysis. Although interactive case study research built on abduction relies on researcher positionality including comprehensive pre-understanding, there is still a need to attend to logical coherence and parsimony in service of quality.^46,68^ Our research team included insiders and outsiders to both context and the theoretical frame, supporting “continuous reflective dialogue” through interpretation, analysis, and writing.^68^ While the focus of this paper is on the change that occurred from 2003 to 2013, one might reasonably inquire as to the sustainability of those efforts a decade later. We have confirmed with a subset of the study participants via email and virtual one-on-one discussion that, despite changes in leadership in parts of the system, the teaching of QI-ICP has been sustained to the time of publishing this article. That said, research into the impacts of leadership changes and significant events, that is, the COVID-19 pandemic, on this transformational change would be useful additions to the literature. A final consideration is that the goal of abductive analysis is to offer hypotheses for future consideration and testing.^69^ We would welcome exploration of the adjusted framework through further research, testing its application in both evaluation of past change and planning of future change.

## Conclusion

The analysis of MU SOM's experience offers key insights into how program transformation in higher education happens in practice. Additionally, the analysis suggests adjustments to a preliminary framework offered by Riaz et al.^14^ as a tool for supporting organizational transformation in health professions education. The framework adjustments make explicit the importance of common purpose and the concept of leadership as an emergent event. They also make it easier to apply the framework to a broader set of organizational contexts, including health professions education. The use of the adjusted framework may help health professions education programs evaluate past change efforts and/or plan for future change.

## Supplemental Material

sj-docx-1-mde-10.1177_23821205251315624 - Supplemental material for A Complex Adaptive Systems Approach to Health Professions Education Transformation: The Case of the University of Missouri-Columbia School of Medicine and an Integrated Quality Improvement-Interprofessional Collaborative Practice Curriculum Supplemental material, sj-docx-1-mde-10.1177_23821205251315624 for A Complex Adaptive Systems Approach to Health Professions Education Transformation: The Case of the University of Missouri-Columbia School of Medicine and an Integrated Quality Improvement-Interprofessional Collaborative Practice Curriculum by Katherine Stevenson, Marcel D’Eon, Linda Headrick and Boel Andersson Gäre in Journal of Medical Education and Curricular Development
